# Efficacy of Chinese herbal medicine in allergic rhinitis: a meta‐analysis

**DOI:** 10.1016/j.bjorl.2026.101769

**Published:** 2026-02-05

**Authors:** Huazhen Zhu, Yongyu Wang, Haoen Zhang, Qi Kang, Lei Xu, Ji Chen, Chen Chen, Jianqing Tao

**Affiliations:** a Shanghai Pudong New Area Pulmonary Hospital, Department of Traditional Chinese Medicine, Shanghai, China; b Shanghai Pudong New Area Pulmonary Hospital, Medical Department, Shanghai, China; c Shanghai Chest Hospital, Research Department, Shanghai, China; d Shanghai Pudong New Area Pulmonary Hospital, Comprehensive Department, Shanghai, China

**Keywords:** Chinese herbal medicine, Allergic rhinitis, Meta‐analysis

## Abstract

**Objective:**

To evaluate the clinical efficacy of Chinese Herbal Medicine (CHM) in treating Allergic Rhinitis (AR) through meta‐analysis.

**Methods:**

A meta‐analysis was conducted using a random effects model to explore the impact of CHM on response rates, nasal symptom scores, quality of life, blood Immunoglobulin E (IgE) levels, and adverse reactions.

**Results:**

A total of 1,326 articles were retrieved, of which 12 studies were included. The meta‐analysis showed that the response rate in the experimental group was higher than in the control group (p < 0.001). The adverse reaction rate was lower in the experimental group compared with the control group (p = 0.003). Subgroup analysis by publication year indicated that studies published in 2024 and 2023 showed lower nasal congestion scores in the experimental group compared with the control group (p < 0.0001); nasal itching scores were lower in the experimental group than in the control group (p < 0.0001). Subgroup analysis by age revealed that the 30+ and 40+ age groups had lower nasal itching scores, sneezing scores and rhinorrhea scores in the experimental group compared with the control group (p < 0.0001). Blood IgE levels were also lower in the experimental group compared with the control group (p = 0.002). Scores for the Rhinoconjunctivitis Quality of Life Questionnaire (RQLQ) were lower in the experimental group (p < 0.0001).

**Conclusion:**

The current evidence suggests that CHM can enhance the efficacy of AR treatment, improve patients’ quality of life, and result in a lower rate of adverse reactions.

**Level of evidence:**

I.

## Highlights

• CHM can enhance the efficacy of AR treatment.
• CHM can improve patients’ quality of life.
• CHM can result in a lower rate of adverse reactions.

## Introduction

Allergic Rhinitis (AR), also known as hay fever, is one of the most common chronic nasal inflammations, primarily caused by an immune response of the nasal mucosa to allergens. The main clinical symptoms of AR include paroxysmal sneezing, rhinorrhea, nasal itching, and nasal congestion, all of which significantly impair the patient’s quality of life.^1^ According to epidemiological surveys, the global prevalence of AR fluctuates between 10% and 40%, with the condition being prevalent in individuals aged 15 to 40.^2^ Moreover, AR is strongly associated with other allergic conditions, such as asthma and eczema, further exacerbating the health and financial burdens on affected individuals.^3^ With the rapid pace of urbanization and the increase in environmental pollution, the incidence of AR is on the rise, making the prevention and treatment of this disease an increasingly urgent issue.^4^

Currently, treatments for AR can be categorized into non‐pharmacological and pharmacological interventions. Non‐pharmacological management includes allergen avoidance, nasal irrigation, and air filtration. However, these methods offer only temporary symptom relief and are often insufficient for long‐term control.^5^ Pharmacological management, such as antihistamines, intranasal corticosteroids, and immunomodulators, can effectively alleviate nasal symptoms. However, long‐term use of these medications may result in side effects, including drowsiness, dry mouth and nose, and, in severe cases, gastrointestinal disturbances and impaired liver and kidney function.^6^ As a result, there is growing interest in finding new therapeutic approaches that can not only provide effective symptom relief but also minimize adverse reactions.

In recent years, the use of complementary and alternative therapies, such as Chinese Herbal Medicine (CHM), for AR treatment has gained increasing attention. As an integral part of traditional Chinese medicine, CHM is considered to have potential efficacy in treating AR due to its diverse pharmacological effects and relatively low side effects.^7^, ^8^, ^9^ However, although several studies suggest that CHM can alleviate AR symptoms to some extent, the results are inconsistent, leaving its exact therapeutic efficacy undetermined. Some studies have shown that CHM can modulate immune responses and inhibit the release of inflammatory mediators, thus significantly improving nasal symptoms in AR patients.^10^^,^^11^ Other studies, however, indicate that the therapeutic outcomes may be influenced by individual differences and variations in herbal combinations.^12^

Therefore, this study conducts a systematic review and meta‐analysis to integrate existing clinical data on the use of CHM in treating AR and evaluate its efficacy and safety. The aim is to resolve the inconsistencies in previous findings and provide more reliable evidence‐based guidance for clinical practice, offering patients with AR more effective targeted treatment options.

## Methods

### Literature retrieval strategy

This study followed the Preferred Reporting Items for Systematic Reviews and Meta‐Analyses (PRISMA 2020) Guidelines^13^ and systematically searched PubMed, Web of Science, Cochrane Library, CNKI, and Wanfang databases. The search period covered the establishment date of each database to September 2024. The search terms included combinations of English and Chinese keywords related to AR and CHM. English search terms included “allergic rhinitis”, “rhinitis, allergic”, “perennial rhinitis”, “pollen allergy”, “Chinese herbal”, “herbal medicine”, “plant extract”, “eastern medicine”, and “alternative medicine”. Chinese search terms included “Chinese herbal medicine”, “allergic rhinitis”, “allergic rhinitis”, and “clinical efficacy”. Additionally, references from included studies and relevant reviews were manually screened to ensure the inclusion of all eligible studies. Two researchers independently conducted the search and screening processes to ensure accuracy and completeness.

### Inclusion and exclusion criteria

Inclusion criteria: (1) Clinical studies published in peer‐reviewed Chinese and English journals, with Chinese studies limited to core journals; (2) Confirmed cases of AR, with no restrictions on age, race, or gender of study participants; (3) Administration of CHM to the experimental group of patients with AR, with no limitations on the form of CHM, including decoctions, tablets, pills, powders, herbal patches, and nasal sprays; (4) The control group could be a blank control, standard treatment, or placebo; (5) The study type should be a prospective clinical study, including Randomized Controlled Trials (RCTs) and cohort studies.

Exclusion criteria: (1) Studies in which the subjects had a history of combined use of CHM or other interventions that could interfere with the efficacy of AR treatment; (2) Studies that were duplicate publications or where data were reused; (3) Studies that could not provide usable data for meta‐analysis; (4) Non‐clinical studies, such as animal experiments or in vitro research.

### Literature screening and data extraction

Two researchers independently screened all the retrieved studies. First of all, studies that did not meet the inclusion criteria were excluded based on the title and abstract. Full texts were then reviewed to determine the final studies for inclusion. In cases of disagreement, a third researcher was consulted to reach a consensus through discussion. Data extraction was also independently conducted by two researchers using standardized data extraction forms. The extracted data included: basic study information (such as the first author, publication year, study region, and study duration), patient demographic data (such as sample size, age, and gender), specific interventions in both the experimental and control groups, duration of the intervention and follow‐up, outcome measures (including primary and secondary outcomes), and bias of risk assessment data. After extraction, all data were reviewed by a third party to ensure accuracy and consistency.

### Definitions of outcome measures

The primary outcome measure in this study was response rate, which refers to the proportion of patients whose nasal symptoms (such as sneezing, rhinorrhea, nasal congestion, and nasal itching) showed significant improvement compared with baseline after treatment. The specific criteria for improvement were based on the definitions provided in each study, including the proportion of patients whose symptoms either significantly lessened or disappeared. The secondary outcome measures included: (1) The Total Nasal Symptom Score (TNSS): This scoring system covers four symptoms ‒ sneezing, nasal itching, rhinorrhea, and nasal congestion ‒ rated based on severity. Changes in the score before and after treatment were used to assess symptom improvement in patients. (2) IgE levels: This refers to changes in the concentration of total or specific IgE in serum, serving as a biomarker to evaluate the effect of CHM on modulating the immune response in patients. (3) Adverse Reaction Rate (ARR): This is the incidence of adverse events occurring during treatment, including gastrointestinal reactions, rashes, and liver or kidney function impairment, used to assess the safety of the treatment. (4) Rhinoconjunctivitis Quality of Life Questionnaire (RQLQ) score: This score reflects the patient’s quality of life, covering daily activities, sleep quality, and emotional status, with a lower score indicating a better quality of life.

### Literature quality assessment

The quality of RCTs was assessed using the Cochrane risk‐of‐bias tool, which evaluates the following aspects: randomization method, allocation concealment, blinding, data integrity, selective reporting, and other potential biases. For cohort studies, the Newcastle‐Ottawa Scale (NOS) was employed, assessing factors such as study group selection, between‐group comparability, outcome assessment, and follow‐up completeness. The maximum score on this scale is 9, and studies scoring ≥7 are considered high‐quality literature.

### Statistical methods

All statistical analyses in this study were performed using the RevMan software (Version 5.4. The Cochrane Collaboration, 2020). Effect sizes for categorical data were expressed as Relative Risk (RR). Continuous data were expressed as Mean Difference (MD) or Standardized Mean Difference (SMD), with 95% Confidence Intervals (95% CI) used to estimate the range of effect sizes. Heterogeneity across studies was assessed using the *Q* test and I² statistic. When the I² value was less than 50% or the p‐value was greater than 0.05, indicating low heterogeneity between studies, a fixed‐effects model was employed to pool effect sizes. If the I² value exceeded 50% or the p‐value was less than 0.05, suggesting significant heterogeneity, a random‐effects model was applied. To further investigate the sources of heterogeneity, sensitivity analyses or subgroup analyses were conducted when necessary. Publication bias was assessed by visually inspecting a funnel plot of the primary outcome, i.e., response rate. If the funnel plot displayed significant asymmetry of effect‐size distribution, publication bias might be present.

## Results

### Literature retrieval and quality assessment results

**Fig. 1:**
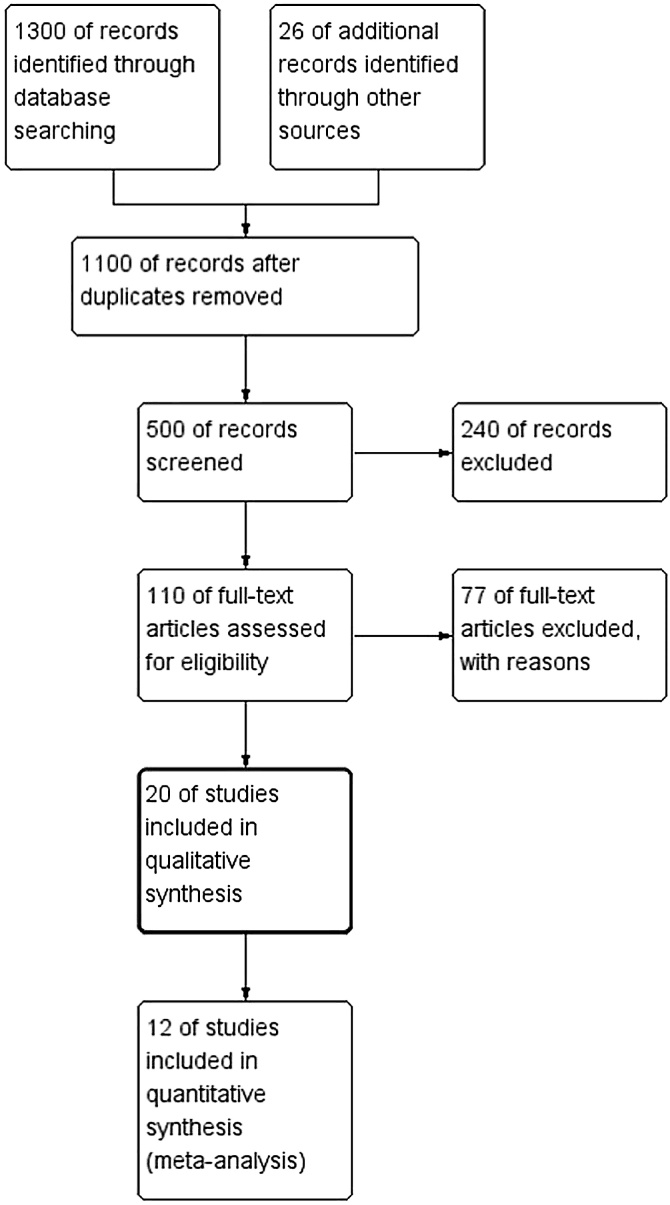
Literature screening process.

**Fig. 2:**
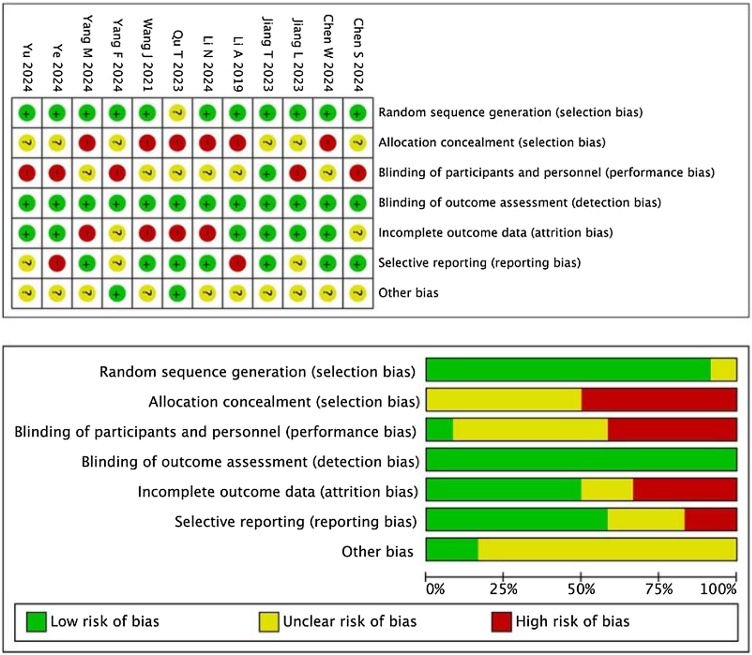
Risk of bias assessment of the 12 included studies.

A total of 1,326 studies were retrieved. After multiple layers of screening, 12 studies were finally included^14^, ^15^, ^16^, ^17^, ^18^, ^19^, ^20^, ^21^, ^22^, ^23^, ^24^, ^25^ (Fig. 1), involving 1,227 patients with AR. Among them, 613 patients were assigned to the control group and 614 to the experimental group. The basic characteristics of the included studies are summarized in Table 1. All studies had a treatment duration of four weeks, except for one study lasting three weeks. The included literature involved diverse study populations from Henan, Zhejiang, Beijing, Guangdong, Shanghai, Heilongjiang, Shandong, Hubei, and Fujian, with sample sizes ranging from 40 to 75 patients, totaling 671 participants. The age ranges in these studies were relatively similar, with an average age of 41.5 ± 5.7 years in the experimental group and 42.7 ± 5.3 years in the control group. The gender distribution was balanced across the studies, although some studies had a slightly higher proportion of female participants. The studies spanned a significant period, from 2016 to 2024, ensuring population diversity and timeliness. The intervention measures used in the studies were varied, primarily involving comparisons between the control and experimental groups. Medications administered to the control groups included loratadine, ebastine, and Bacillus Calmette‐Guerin polysaccharide nucleic acid, while the experimental groups were treated with different Chinese herbal formulas, such as Zhenwu Decoction, lung‐warming and nose‐unblocking decoction, Xiaoqinglong Decoction, and Xiangju (wild chamomile) tablets. Each herbal formula had unique compositions. For example, Zhenwu Decoction consisted of CHMs like prepared common monkshood root (*Radix Aconiti Lateralis Preparata*), fresh ginger (*Rhizoma Zingiberis Recens*), white atractylodes (*Rhizoma Atractylodis Macrocephalae*), and white peony (*Radix Paeoniae Alba*). The results of the literature quality assessment are shown in Fig. 2.

**Table 1:** Basic characteristics of the 12 included studies.

First author & publication year	Region	Sample size (n)		Age		Gender (n)		Study period
		Control group	Experimental group	Control group	Experimental group	Male	Female	
Chen WM, 2024	Henan	60	60	42.7 ± 5.3	41.5 ± 5.7	82	38	June 2022 to May 2023
Ye XH, 2024	Zhejiang	61	62	43.5 ± 9.25	44.29 ± 9.42	72	51	September 2020 to June 2022
Yang ML, 2024	Beijing	42	42	37.55 ± 7.23	37.64 ± 7.30	45	39	June 2021 to December 2022
Li N, 2024	Guangdong	43	43	42.65 ± 4.70	42.73 ± 4.81	52	34	April 2021 to March 2022
Jiang TT, 2023	Shanghai	50	50	33.26 ± 7.63	32.43 ± 8.38	53	47	January 2021 to September 2022
Yang F, 2024	Heilongjiang	40	40	35.61 ± 4.67	35.31 ± 4.23	35	45	November 2021 to December 2022
Yu XY, 2024	Shandong	75	75	42.18 ± 13.76	42.59 ± 11.52	79	71	August 2021 to May 2023
Qu TF, 2023	Zhejiang	51	51	45.17 ± 5.24	45.25 ± 5.18	53	49	December 2020 to December 2022
Jiang LY, 2023	Guangdong	50	50	9.87 ± 1.05	9.45 ± 1.16	60	40	December 2021 to November 2022
Wang J, 2021	Hubei	53	53	31.24 ± 2.35	31.19 ± 2.39	41	65	June 2018 to June 2019
Chen S, 2024	Fujian	42	42	6.30 ± 1.14	6.61 ± 1.19	49	35	October 2022 to March 2023
Li A, 2019	Guangdong	46	46	39.5 ± 8.2	41.35 ± 8.4	48	44	March 2016 to March 2017

First author & publication year	Treatment duration (weeks)	Intervention measure		CHM composition	Outcome measure
		Control group	Experimental group		
Chen WM, 2024	4 weeks	Mometasone furoate plus levocetirizine	Zhenwu Decoction	Common monkshood root (Radix Aconiti Lateralis Preparata) 10 g, fresh ginger (Rhizoma Zingiberis Recens) 10 g, white atractylodes (Rhizoma Atractylodis Macrocephalae) 20 g, and white peony (Radix Paeoniae Alba) 10 g, blond magnolia flower (Flos Magnoliae) 10 g, xanthium fruit (Fructus Xanthii) 9 g, Chinese wild ginger root (Radix et Rhizoma Asari) 3 g	(1) (2) (3) (4)
Ye XH, 2024	4 weeks	Loratadine	Treatment administered to the control group + Lung‐warming nose‐unblocking decoction	Chinese ephedra (Herba Ephedrae) 10 g, dried ginger (Rhizoma Zingiberis) 10 g, cassia twig (Ramulus Cinnamomi) 6 g, aconite root (Radix Aconiti) 5 g, Chinese wild ginger root (Radix et Rhizoma Asari) 5 g, xanthium fruit (Fructus Xanthii) 12 g, blond magnolia flower (Flos Magnoliae) 12 g, astragalus root (Radix Astragali) 10 g, walnut (Semen Juglandis) 10 g, cicada moulting (Periostracum Cicadae) 10 g, smoked plum (Fructus Mume) 10 g, licorice (Radix et Rhizoma Glycyrrhizae) 6 g	(1) (2) (3) (5)
Yang ML, 2024	4 weeks	Loratadine	Treatment administered to the control group + Xiaoqinglong Decoction	Cassia twig (Ramulus Cinnamomi) 10 g, white peony root (Radix Paeoniae Alba) 10 g, Chinese magnolivine fruit (Fructus Schisandrae Chinensis) 10 g, dried ginger (Rhizoma Zingiberis) 6 g, pinellia tuber (Rhizoma Pinelliae) 10 g, cannabis (Cannabis Sativa) 10 g, licorice (Radix et Rhizoma Glycyrrhizae) 6 g, aconite root (Radix Aconiti) 3 g	(1) (2) (4) (5)
Li N, 2024	4 weeks	Desloratadine citrate disodium tablets	Treatment administered to the control group + Xiangju (wild chamomile) tablets	Platycary fruit (Fructus Platycarya Strobilacea), common self‐heal fruit‐spike (Spica Prunellae), wild chrysanthemum flower (Flos Chrysanthemi), raw astragalus root (Radix Astragali), blond magnolia flower (Flos Magnoliae), siler (Radix Saposhnikoviae), angelica root (Radix Angelicae Dahuricae), licorice (Radix et Rhizoma Glycyrrhizae), Szechuan lovage rhizome (Rhizoma Ligusticum)	(1) (2) (3) (4) (5)
Jiang TT, 2023	4 weeks	Lung‐warming qi‐replenishing formula ×10%	Lung‐warming qi‐replenishing formula ×100%	Astragalus root (Radix Astragali) 30 g, white atractylodes rhizome (Rhizoma Atractylodis Macrocephalae) 10 g, siler (Radix Saposhnikoviae) 10 g, blond magnolia flower (Flos Magnoliae) 10 g, angelica root (Radix Angelicae Dahuricae) 10 g, Chinese wild ginger root (Radix et Rhizoma Asari) 3 g, scutellaria root (Radix Scutellariae) 5 g, honey‐fried cannabis (Cannabis Sativa Praeparata cum Melle) 3 g, licorice (Radix et Rhizoma Glycyrrhizae) 6 g	(1) (2) (3) (5)
Yang F, 2024	4 weeks	Ebastine	Treatment administered to the control group + Cassia Twig Plus Astragalus Root Decoction	Cassia twig (Ramulus Cinnamomi) 15 g, dried ginger rhizome (Rhizoma Zingiberis) 10 g, licorice (Radix et Rhizoma Glycyrrhizae) 9 g, astragalus root (Radix Astragali) 20 g, xanthium fruit (Fructus Xanthii) 15 g, blond magnolia flower (Flos Magnoliae) 10 g	(1) (2) (3) (5)
Yu XY, 2024	4 weeks	Tranilast Capsules	Treatment administered to the control group + Biyuan Tongqiao Granules	Blond magnolia flower (Flos Magnoliae), xanthium fruit (Fructus Xanthii), ephedra herb (Herba Ephedrae), angelica root (Radix Angelicae Dahuricae), mint (Herba Menthae), Sichuan lovage (Ligusticum Sinense), scutellaria root (Radix Scutellariae), forsythia (Fructus Forsythiae), wild chrysanthemum flower (Flos Chrysanthemi), snakegourd root (Radix Trichosanthis), rehmannia root (Rehmannia Glutinosa), Danshen root (Salvia Miltiorrhiza), poria (Poria Cocos), licorice (Radix et Rhizoma Glycyrrhizae)	(1) (2) (4) (5)
Qu TF, 2023	4 weeks	Ebastine	Treatment administered to the control group + Qi‐replenishing allergy‐resolving decoction	Honey‐fried astragalus root (Radix Astragali Praeparata cum Melle) 12 g, blond magnolia flower (Flos Magnoliae) 10 g, thorowax root (Radix Bupleuri) 10 g, smoked plum (Fructus Mume) 10 g, siler (Radix Saposhnikoviae) 6 g, white peony root (Radix Paeoniae Alba) 6 g, cassia twig (Ramulus Cinnamomi) 6 g, angelica root (Radix Astragali) 6 g, licorice (Radix et Rhizoma Glycyrrhizae) 3 g	(1) (2) (3) (4)
Jiang LY, 2023	3 weeks	Loratadine	Treatment administered to the control group + Sanfeng Tongqiao Pills	Scutellaria root (Radix Scutellariae), catnip (Nepeta Cataria), notopterygium root (Rhizoma et Radix Notopterygii), Chinese wild ginger root (Radix et Rhizoma Asari)	(1) (2) (3) (4)
Wang J, 2021	4 weeks	Bacillus Calmette‐Guerin polysaccharide nucleic acid + loratadine tablets + budesonide nasal spray	Treatment administered to the control group + Xinyi Decoction	Blond magnolia flower (Flos Magnoliae) 30 g, chamomile (Chrysanthemum Lavandulifolium) 30 g, angelica root (Radix Angelicae Dahuricae) 30 g, hogfennel root (Radix Peucedani) 30 g, Szechuan lovage rhizome (Rhizoma Ligusticum) 30 g, mint leaf (Herba Menthae) 30 g, gypsum (Gypsum Fibrosum) 30 g, white atractylodes rhizome (Rhizoma Atractylodis Macrocephalae) 30 g, hoelen (Poria Rubra) 30 g, raw dried rhemannia (Radix Rehmanniae Recens) 30 g, tangerine pericarp (Pericarpium Citri Reticulatae) 30 g, licorice (Radix et Rhizoma Glycyrrhizae) 60 g, grassleaf sweetflag rhizome (Rhizoma Acori Tatarinowii) 10 g, xanthium fruit (Fructus Xanthii) 10 g, rubarb (Radix et Rhizoma Rhei) 20 g	(1) (2) (3) (4) (5)
Chen S, 2024	4 weeks	Montelukast Chewable Tablets	Treatment administered to the control group + Healthy qi‐reinforcing and orifices‐opening decoction	Fishwort (Herba Houttuyniae) 30 g, apricot kernel (Semen Armeniacae Dulce) 10 g, astragalus root (Radix Astragali) 30 g, white atractylodes rhizome (Rhizoma Atractylodis Macrocephalae) 10 g, xanthium fruit (Fructus Xanthii) 10 g, gypsum (Gypsum Fibrosum) 30 g, ephedra herb (Herba Ephedrae) 10 g, blond magnolia flower (Flos Magnoliae) 10 g, siler (Radix Saposhnikoviae) 10 g, honey‐fried licorice (Radix et Rhizoma Glycyrrhizae Praeparata cum Melle) 6 g, angelica root (Radix Angelicae Dahuricae) 10 g, Chinese wild ginger root (Radix et Rhizoma Asari) 3 g	(1) (3) (4) (5)
Li A, 2019	4 weeks	Cetizizine Hydrochloride Drops	Treatment administered to the control group + Yuping Cang’er Mixture	Astragalus root (Radix Astragali) 15 g, white atractylodes rhizome (Rhizoma Atractylodis Macrocephalae) 15 g, siler (Radix Saposhnikoviae) 10 g, xanthium fruit (Fructus Xanthii) 9 g, scutellaria root (Radix Scutellariae) 10 g, blond magnolia flower (Flos Magnoliae) 10 g, angelica root (Radix Angelicae Dahuricae) 10 g, Szechuan lovage rhizome (Rhizoma Ligusticum), mint leaf (Herba Menthae) 10 g, Chinese wild ginger root (Radix et Rhizoma Asari) 5 g, earthworm (Pheretima Posthuma) 15 g, licorice (Radix et Rhizoma Glycyrrhizae) 6 g	(1) (2) (3)

(1) Response rate; (2) TNSS score; (3) IgE level; (4) ARR; (5) RQLQ score.

### Meta‐analysis results

#### Response rate

**Fig. 3:**
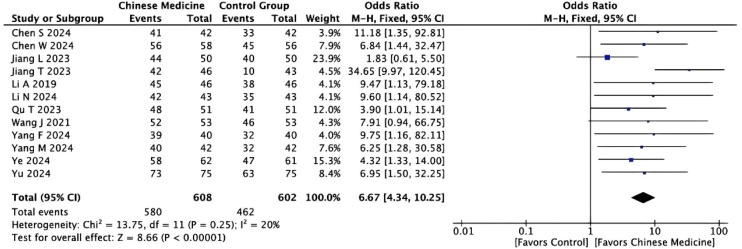
Forest plot comparing the response rate between the Chinese medicine group and the control group. M-H, Mantel-Haenszel method; CI, Confidence Interval.

A total of 12 studies compared the response rates between the two groups. As the studies were homogeneous (I² = 20%, p < 0.001), a fixed‐effects model was applied for the meta‐analysis. The results indicated that the experimental group had a higher response rate than the control group (MD = 6.67, 95% CI [4.34, 10.25]) (Fig. 3).

A funnel plot based on the response rates reported in the included studies was generated to assess potential publication bias. As shown in Figure S1, the funnel plot displays an asymmetric distribution, suggesting the possibility of publication bias.

ARR

**Fig. 4:**
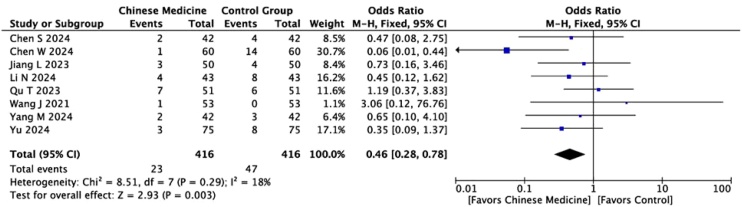
Forest plot comparing the Adverse Reaction Rate (ARR) between the Chinese medicine group and the control group. M-H, Mantel-Haenszel method; CI, Confidence Interval.

Nine studies compared the ARRs between the two groups. Given the homogeneity of the studies (I² = 18%, p = 0.003), a fixed‐effects model was used for this meta‐analysis. The results showed that the ARR in the experimental group was lower than in the control group (MD = 0.46, 95% CI [0.28, 0.78]) (Fig. 4).

We found that the study by Chen et al. (2024) seemed to strongly bias the overall results in favor of TCM. Therefore, we conducted a sensitivity analysis to assess the impact of the study by Chen et al. (2024), which contributed a substantial weight to the analysis. After excluding this study, the pooled results remained statistically significant (MD = 0.54, 95% CI [0.37, 0.79], p = 0.001), continuing to indicate that the incidence of adverse reactions in the CHM group was lower than that in the control group. This confirms that the overall findings are robust and not solely dependent on a single study.

#### TNSS

**Fig. 5:**
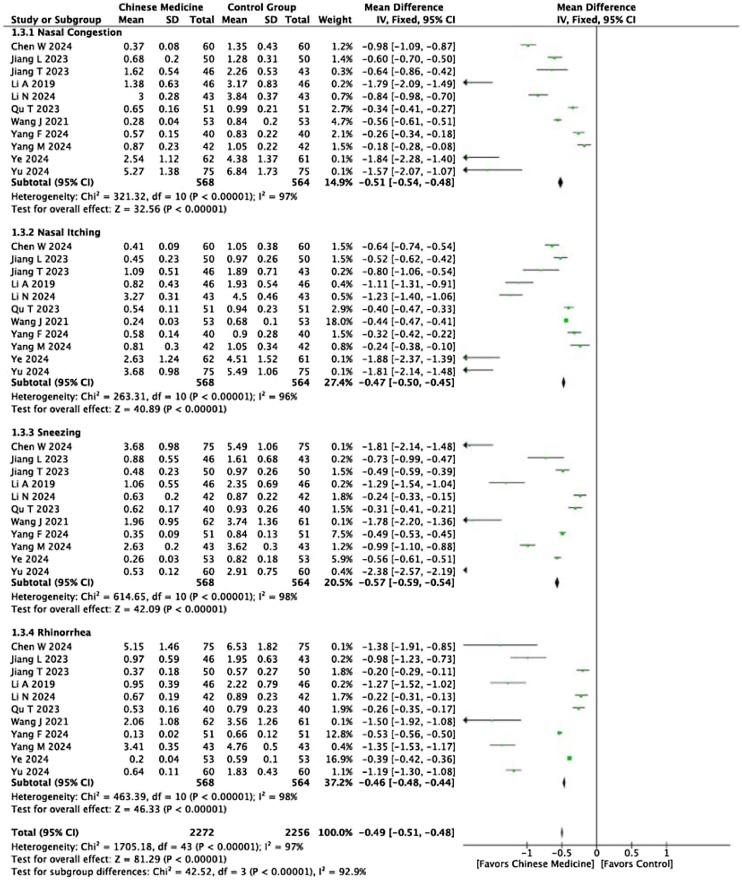
Meta‐analysis forest plot of TNSS scores.

Eleven studies analyzed the TNSS scores in both groups. As shown in Fig. 5, the nasal congestion score in the experimental group is lower than in the control group (I² = 97%, p < 0.0001, MD = −0.51, 95% CI [−0.54, −0.48]). The subgroup analysis by publication year revealed that clinical studies published in 2024 and 2023 reported significantly lower nasal congestion scores in the experimental group compared with the control group (I² = 0%, p < 0.0001, MD = −1.72, 95% CI [−2.05, −1.39] for 2024 studies; I² = 0%, p < 0.0001, MD = ‐0.60, 95% CI [−0.70, −0.50] for 2023 studies). Additionally, the nasal itching score was lower in the experimental group than in the control group (I² = 96%, p < 0.0001, MD = −0.77, 95% CI [‐0.93, ‐0.61]). Subgroup analyses by publication year and age were performed for studies published in 2024, focusing on participants aged 30+ and 40+. The nasal itching scores in the experimental group were significantly lower than in the control group (I² = 0%, p < 0.0001, MD = −0.29, 95% CI [−0.37, −0.21] for or the 30+ age group; I² = 0%, p < 0.0001, MD = −0.60, 95% CI [−0.70, −0.50] for the 40+ age group). Similarly, the sneezing scores in the experimental group were also significantly lower compared with the control group (I² = 8%, p < 0.0001, MD = −0.27, 95% CI [−0.34, −0.21] for the 30+ age group; I² = 0%, p < 0.0001, MD = −1.80, 95% CI [−2.06, −1.54] for the 40+ age group). For the rhinorrhea scores, the experimental group also showed significant improvements over the control group (I² = 0%, p < 0.0001, MD = −1.37, 95% CI [−1.53, −1.22] for the 30+ age group; I² = 0%, p < 0.0001, MD = −0.24, 95% CI [−0.30, −0.18] for the 40+ age group).

#### Serum IgE levels and RQLQ scores

**Fig. 6:**
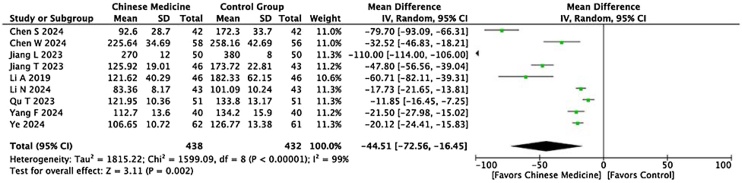
Meta‐analysis forest plot of serum IgE levels.

**Fig. 7:**
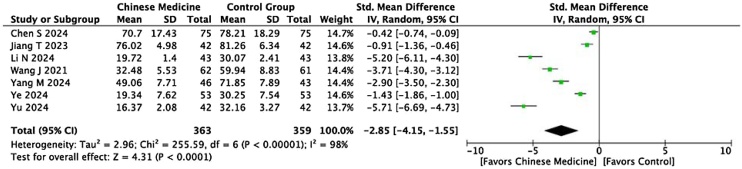
Forest plot comparing the Rhinoconjunctivitis Quality of Life Questionnaire (RQLQ) scores between the Chinese medicine group and the control group. SMD, Standardized Mean Difference; CI, Confidence Interval; IV, Inverse Variance method. SD, Standard Deviation.

A total of 10 studies compared serum IgE levels between the two groups. The meta‐analysis showed that serum IgE levels in the experimental group were significantly lower than in the control group (I² = 99%, p = 0.002, MD = −44.51, 95% CI [−72.56, −16.45]) (Fig. 6). Additionally, 8 studies compared the RQLQ scores between the two groups. With a higher RQLQ score indicating a greater negative impact on quality of life, the score in the experimental group was significantly lower than in the control group (I² = 98%, p < 0.0001, MD = −2.85, 95% CI [−4.15, −1.55]) (Fig. 7). However, substantial heterogeneity was observed, which could be attributed to the wide range of patient ages (from children to young adults), variations in units of IgE measurement, and inconsistency in the RQLQ scoring tools used across studies. This highlights the importance of standardizing measurement units and scoring criteria to reduce heterogeneity in future research.

## Discussion

This study systematically evaluated the clinical efficacy and safety of CHM in treating AR. By integrating data from 12 relevant studies, encompassing clinical data from 1,227 patients, we found that CHM demonstrated significant advantages in alleviating nasal symptoms, particularly in improving treatment efficacy and reducing adverse reactions. These findings provide new evidence for the application of CHM in the treatment of AR, suggesting that CHM not only holds an important position in traditional medicine but also warrants greater attention from the modern medical community.

Our findings are consistent with previous literature, highlighting the potential of CHM in improving the symptoms of patients with AR. Numerous studies have shown that CHM can not only modulate immune responses and inhibit the release of inflammatory factors but also improve patients’ overall quality of life.^26^ Specifically, formulas such as Xiaoqinglong Decoction and Zhenwu Decoction, with their unique pharmacological mechanisms, can effectively relieve typical symptoms such as nasal congestion, rhinorrhea, and sneezing. The active ingredients in these formulas, including cassia twig (*Ramulus Cinnamomi*), Chinese ephedra (*Herba Ephedrae*), and white peony (*Radix Paeoniae Alba*), are known for their properties of warming and dispersing cold, as well as harmonizing qi and blood, which are critical in improving nasal symptoms.^27^^,^^28^ These findings not only demonstrate the efficacy of CHM in AR treatment but also promisingly contribute to the comprehensive management of allergic diseases.

Furthermore, we also attempted to elucidate the potential mechanisms by which CHM treats AR. CHM often contains multiple active ingredients that can act on the body through various pathways, thereby modulating the immune system.^29^ For instance, some components of CHM possess antioxidant and anti‐inflammatory properties, which can relieve symptoms by reducing the inflammatory response of the nasal mucosa. Specifically, ingredients such as Baikal skullcap (*Radix Scutellariae*) and forsythia (*Fructus Forsythiae*) can inhibit the release of inflammatory mediators, reduce local tissue edema, and enhance the immune function of the upper respiratory tract.^30^ In addition, certain herbs like *Artemisia ordosica Krasch* potentially enhance tolerance to allergens by inhibiting IgE production and reducing the occurrence of allergic reactions.^31^ For instance, studies have shown that some CHM ingredients can regulate T‐cell function, promote Th1‐type immune responses, and effectively counteract Th2‐cell‐mediated allergic reactions.^32^ These mechanisms provide theoretical support for the application of CHM in AR treatment via multiple pathways.

The main strength of the present study lies in its systematic evaluation of the efficacy and safety of CHM in treating AR. First, the study included multiple high‐quality RCTs, ensuring the reliability of the data and the validity of the results. Second, strict inclusion criteria and rigorous statistical methods were employed, which helped reduce bias and enhance the generalizability of the conclusions. Moreover, this study not only focused on the improvement of nasal symptoms using CHM but also assessed the impact of CHM on adverse reactions, further enriching the treatment options for patients with AR. Although the results of this study demonstrated the positive role of CHM in treating AR, several limitations exist. First, the number of studies included in the study is relatively small. Although the sample size reached 1,227 cases, the variations in intervention measures and drug combinations across studies may have affected the consistency and generalizability of the results. For example, differences in drug dosage, treatment duration, and baseline characteristics of patients in different studies could have led to variability in clinical outcomes. This issue needs to be addressed in future research to enhance the credibility of our findings. Second, the present study primarily relies on existing clinical trial data and lacks large‐scale, long‐term prospective studies further verifying the efficacy and safety of CHM. Moreover, another limitation of this study is that the included primary studies did not report in detail the sensitization status of patients to specific allergens (such as house dust mites, pollen, mold, etc.). As is well known, the spectrum of allergens in allergic rhinitis has significant regional characteristics. Since all the trials included in this study were conducted in China, the main allergens may differ from those in other regions of the world. Therefore, the results of this study are mainly applicable to populations with similar allergen exposure backgrounds (such as those sensitized to common allergens in China). Future studies should report the allergen sensitization profiles of patients in detail to allow for more refined subgroup analyses to explore whether there are differences in the efficacy of traditional Chinese medicine therapy for patients with different types of allergens. Additionally, individual differences and the specific mechanisms of action of CHM require further exploration, providing important directions for future research. In‐depth studies on the effects of specific CHM, both alone and in combination with other treatments, will help us better understand the clinical effects of CHM.

In addition, it is necessary to explore the balance between the standardization of Traditional Chinese Medicine (TCM) formulas and individualized treatment. Unlike single-molecule compounds, the core of TCM treatment for allergic rhinitis lies in “syndrome differentiation and treatment”, which means adjusting the formula according to the individual patient's symptoms and signs. This leads to some differences in the specific composition and dosage of the intervention measures. Such an individualized approach is the source of the clinical advantage of traditional Chinese medicine, but it also poses challenges to the standardized interpretation of research results and their reproducibility on a global scale. What this meta-analysis has confirmed is the effectiveness of the overall strategy of “using TCM formulas under the guidance of TCM theory”. Future studies should focus on better reporting the rules of syndrome differentiation and formula adjustment, and strengthen the research on the core active components of the formula and their mechanisms of action. In this way, it is possible to both maintain the essence of individualized TCM treatment and provide more scientific evidence for the clinical efficacy of TCM that is easier for the international community to understand and accept, ultimately promoting its standardized application and dissemination worldwide.

## Conclusion

Existing evidence suggests that oral administration of CHM can clinically improve the response rate, reduce adverse reactions, and alleviate nasal symptoms in patients with AR. However, given the quality of the included studies, our study results still require further confirmation by higher‐quality, multicenter, large‐scale RCTs.

## ORCID ID

Huazhen Zhu: 0009-0006-7872-9442

Yongyu Wang: 0009-0000-4493-6087

Haoen Zhang: 0009-0007-4520-0507

Qi Kang: 0009-0009-3702-2135

Lei Xu: 0009-0004-6891-1633

Ji Chen: 0009-0004-7533-7238

Chen Chen: 0009-0003-4782-6600

Jianqing Tao: 0009-0009-8787-4379

## CRediT authorship contribution statement

Chen C and Tao JQ conceived of the study, and Zhu HZ, Wang YY, Zhang HE, Kang Q, Xu L and Chen J participated in its design and data analysis and statistics and Zhu HZ and Wang YY helped to draft the manuscript. All authors read and approved the final manuscript.

## Consent for publication

Not applicable.

## Funding

This study was supported by Construction Project of Famous TCM Doctor Studio in Pudong New Area, Shanghai (Project No.: PDZY-2025-0719) and Pudong New Area Traditional Chinese Medicine Inheritance and Innovation Development Demonstration Pilot Project: Flagship Hospital of Traditional Chinese and Western Medicine Collaboration (nº YC-2023-0402). Funding agencies did not play a role in study design, data collection, analysis and interpretation, and manuscript writing.

## Ethics approval and consent to participate

An ethics statement is not applicable because this study is based exclusively on published literature.

## Availability of data and materials

All data generated or analysed during this study are included in this article. Further enquiries can be directed to the corresponding author.

## Declaration of competing interest

All of the authors had no any personal, financial, commercial, or academic conflicts of interest separately.
