# Supplementary material for: Efficacy of Chinese herbal medicine in allergic rhinitis: a meta‐analysis
Source: Braz J Otorhinolaryngol. 2026 Feb 5;92(2):101769. doi: 10.1016/j.bjorl.2026.101769 (PMC12906175; doi:10.1016/j.bjorl.2026.101769)

**BJORL-D-25-00160_Supplementary Material**

**Figure S1** Funnel plot of publication bias based on post‐treatment response rates.


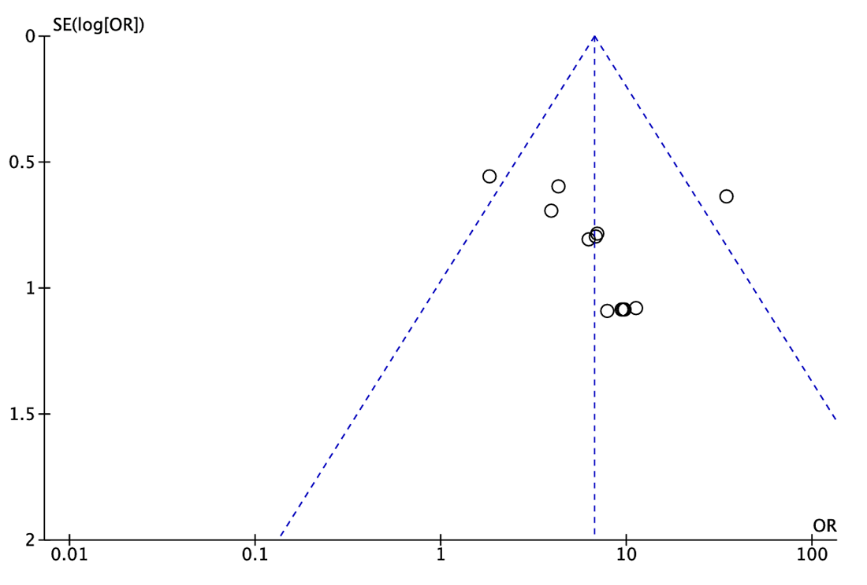

Supplement: Supplementary file 1 [file mmc1.docx]
